# Structural properties and anti-inflammatory activity of purified polysaccharides from *Hen-of-the-woods* mushrooms (*Grifola frondosa*)

**DOI:** 10.3389/fnut.2023.1078868

**Published:** 2023-02-07

**Authors:** Xiaoyi Liu, Shuai Chen, Huijuan Liu, Jiao Xie, K. M. Faridul Hasan, Qibing Zeng, Shaofeng Wei, Peng Luo

**Affiliations:** ^1^The Key Laboratory of Environmental Pollution Monitoring and Disease Control, Ministry of Education, Department of Nutrition and Food Hygiene, School of Public Health, Guizhou Medical University, Guiyang, China; ^2^Department of Biochemistry and Molecular Biology, College of Basic Medical Sciences, Guizhou Medical University, Guiyang, China; ^3^Simonyi Károly Faculty of Engineering, University of Sopron, Sopron, Hungary

**Keywords:** *Grifola frondosa* polysaccharides, ultrasound-assisted extraction, structural properties, ulcerative colitis, anti-inflammatory activity

## Abstract

*Grifola frondosa* is an edible medicinal mushroom that has been proven to have a variety of health benefits. The main active ingredients of this mushroom are polysaccharides. In this study, ultrasonic-assisted extraction was used to obtain crude Grifola frondosa polysaccharides (GFPs). Then, purified GFP was obtained after purification. The optimum extraction conditions were an extraction time of 71 min, an extraction temperature of 90°C in a solid-to-liquid ratio of 1:37 g/mL, and an ultrasonic power of 500 W. GFP was purified using DEAE-52 and Sephadex G-100. The structural characterization of GFP was performed using Fourier transform infrared spectroscopy (FT-IR), X-ray diffraction (XRD), ion chromatography (IC), and ultraviolet (UV) visible photometry. The morphology of GFP was analyzed by scanning electron microscopy (SEM), thermogravimetric differential scanning calorimetry (TG-DSC), and Congo red testing. In addition, the administration of GFP in oxazolone (OXZ)-induced ulcerative colitis (UC) in mice was found to prevent weight loss. Different doses of GFP (80, 160, and 320 mg/kg body weight) were used, and sulfapyridine (SASP) was used as a positive control (370 mg/kg body weight) for the treatment of OXZ-induced UC. After treatment, the mice were killed, and blood and colon tissue samples were collected. GFP was found to prevent decreases in colon length and the levels of leukocytes, platelets, and neutrophils in UC mice. Moreover, GFP also decreased the expression of pro-inflammatory cytokines [tumor necrosis factor (TNF)-α and interleukin (IL)-1 β], increased IL-10, and reduced colon injury in UC mice. The results showed that Under these conditions, the predicted polysaccharide yield was 21.72%, and the actual extraction rate was 21.13%. The polysaccharide composition (molar ratio) was composed of fucose (0.025), glucosamine hydrochloride (0.004), galactose (0.063), glucose (0.869), and mannose (0.038). GFP was also found to have a typical absorption peak, and the GFP extracted using the ultrasound-assisted extraction protocol was mainly β-glucan. These results indicate that ultrasound-assisted extraction of GFP could reduce OXZ-induced intestinal inflammation as a promising candidate for the treatment of UC, with the potential for development as a food supplement to improve intestinal diseases.

## 1. Introduction

*Grifola frondosa* is a medicinal and edible fungus, commonly known as “*Maitake*” and “*Hen-of-the-woods*” (1). GF is rich in active ingredients including polysaccharides, polyphenols, steroids, alkaloids, and trace elements (2). Among these, GFP is one of its main active components. Previous studies have found that the structure of GFP was composed of β-glucans, α-D-glucan, heteroglycans, D-grade, and MZ-grade (3–5), which exert various functions. FangMa, and Zhao et al. demonstrated that GFP could enhance immunity (4, 6, 7). Similarly, Mao and Masuda et al. found that GFP had significant anti-tumor activity (3, 8). Wang et al. reported that β-glucan in GFP could significantly inhibit the cytopathic effect (CPE) induced by herpes simplex virus type I (9). In addition, GFP had been shown to have hypoglycemic (10), anti-hyperlipidemia (11), and anti-radiation effects (12), as well as protecting the gastric mucosa and liver (13). Therefore, GFP has often been referred to as a biological response modifier (BRM) (14). The structure of polysaccharides may determine their biological functions (15), suggesting that the elucidation of the structure of polysaccharides could help researchers improve their understanding of their medicinal functions (16, 17).

Ulcerative colitis (UC) is a chronic inflammatory disease, with only the colonic mucosa as the onset site. It is a long-term disease with an unpredictable healing period that is characterized by alternating periods of deterioration and remission (18). One study found that the annual incidence of UC was 12.6/100,000 people in the United Kingdom (19). It is worth noting that the prevalence of UC appears to be on the rise, with the latest figures for Lothians in the United Kingdom showing a prevalence of 432/100,000 as of 2020 (20). In addition, the study also found that the global incidence of UC has increased sharply in recent years, bringing a huge medical and economic burden to the world (21, 22). To date, the etiology and pathogenesis of UC are not yet fully understood, and there is currently no effective prevention or treatment method (23). Clinically, immunosuppressants (adalimumab and infliximab) and anti-inflammatory drugs (corticosteroids and 5-aminosalicylic acid) are commonly used to treat UC (24). However, these drugs often have some limitations and adverse reactions (25). Therefore, it is necessary to identify natural and non-toxic drugs for the treatment of UC. Presently, plant polysaccharides such as *Astragalus membranaceus*, *Codonopsis pilosula* (26), Shaoyao decoction (27), and *Scutellaria baicalensis Georgi* (28) have been used to treat colitis and have proven to be effective. However, very few studies have been conducted on the treatment of colitis using purified GFP.

The anti-inflammatory effect mediated by GFP has long been the focus of natural product research (29, 30). However, the extraction, purification, and structural characterizations of GFP as a starting point to explore its anti-colitis effects have not yet been reported. A basic understanding of the structure and biological activity of these natural polysaccharides is essential for the future of medicine and other industries worldwide. Thus, the purpose of this study was to identify an optimal method for the extraction and purification of GFP and to evaluate the anti-inflammatory activity of GFP in mice with UC.

## 2. Materials and methods

### 2.1. Materials

#### 2.1.1. Samples

Samples were obtained from the fungal medicine industrial base in Qinglong county, Guizhou Province, China. The cultivation conditions (temperature, humidity, and pH) were strictly controlled. The samples were selected by experts and freeze-dried after cleaning. Thereafter, the samples were powdered using a pulverizer for further analysis.

#### 2.1.2. Instruments and reagents

##### 2.1.2.1. Instruments

The analytical instruments used in this study were as follows: multi-purpose constant temperature ultrasonic extraction machine (TL-1000CT; Jiangsu Tianling Instrument Co., Ltd., China), rotary evaporator (RE-52; Shanghai Yarong Biochemical Instrument Factory, China), constant temperature shaker (TS-111B; Shanghai Tiancheng Experimental Instrument Manufacturing Co., Ltd., China), vacuum dryer (DZF-6050; Shanghai Qixin Scientific Instrument Co., Ltd., China), microplate reader (Thermo Fisher Scientific Co., Ltd., China), constant flow pump (BT-100D; Shanghai Qingpu Huxi Instrument Factory, China), high-speed refrigerated centrifuge (TDL-5000bR; Shanghai Anting Scientific Instrument Factory, China), nitrogen blower (UGC-24M; Lichen Technology, China), automatic blood analyzer (Shenzhen Mindray Medical Co., Ltd., China), and electric constant temperature blast drying box (101-1BS; Lichen Technology, China).

##### 2.1.2.2. Reagents

The reagents used in this study were as follows: anhydrous ethanol (Tianjin Zhiyuan Chemical Reagent Co., Ltd., China); Coomassie Brilliant Blue G-250 Solution, Polyamide, Sephadex G-100 (Beijing Soleibo Technology Co., Ltd., China); DEAE Cellulose DE-52 (Beijing Soleibao Technology Co., Ltd., China); trifluoroacetic acid (ACROS A0356762 139725000 AR), 50% sodium hydroxide solution (Z21E036 33382 GR) (Alfa Aesar, China); sodium acetate (191126 059326 GR) (Thermo Fisher Scientific, China), single sugar standard (Bo Rui Sugar Biological Co., Ltd., China); IFN-r Mouse Uncoated ELISA Kit, IL-4 Mouse Uncoated ELISA Kit, and Mouse IgG ELISA Kit (LinkBio, China).

### 2.2. Methods

#### 2.2.1. Extraction and purification of crude GFP

##### 2.2.1.1. Extraction process

*Grifola frondosa* powder was added to ultrapure water according to the material-to-liquid ratio reported by Yao (1). For ultrasound-assisted extraction of crude GFP, the mixture was placed in an ultrasonic constant temperature extractor to obtain GFP. Extraction was performed with a 1:3 ratio of the filtrate to absolute ethanol, mixed well, and placed in a 40aced in thator overnight for alcohol precipitation (12 h). Then, the mixture was centrifuged at 2,504 × g for 20 min. The supernatant was discarded and the precipitate was vacuum dried at 60°C. Next, 1.00 g of the dried sample was dissolved in 1 mL of pure water by ultrasonication. Finally, the polysaccharide content was determined using the phenol-sulfuric acid method, and the extraction rate was calculated using the mass ratio method.

##### 2.2.1.2. Standard curve creation and polysaccharide detection

To prepare 1 mL of polysaccharide standard, 1 mL of 5% phenol-water solution and 5 mL of concentrated H_2_SO_4_ were used. Tube 0 was used as a blank tube after measuring the absorbance at 490 nm. A standard curve was obtained (y = 0.0047x + 0.0178, *R*^2^ = 0.9994) with a linearity range of 0–800 μg/mL. The polysaccharide content in the samples was detected based on the detection method of the standard.

##### 2.2.1.3. Optimization of crude GFP extraction method

The extraction time, extraction temperature, solid-to-liquid ratio, and ultrasonic power were selected as the influencing factors of the polysaccharide extraction rate, and a single-factor experiment was carried out.

According to the above single-factor test results, three levels of each treatment condition, extraction time, extraction temperature, solid-to-liquid ratio, and ultrasonic power were determined. Using the GFP extraction rate as the response index, the results of the response surface method (RSM) were determined, and the optimal method was analyzed using Design-Expert 13.

##### 2.2.1.4. Purification of crude GFP

(1) Polyamide for the first purification

As described by Hu et al. (31) and Li and Li (32), 0.5, 1.0, 1.5, 2, and 2.5 g of polyamide were soaked in 95% ethanol for 2 h. Next, the ethanol was removed, and the sample was washed with distilled water three times and soaked in distilled water for 2 h, followed by ultrasonication and filtration. The polyamides were then added to 100 mL of GFP solution at the same concentration to prepare polyamides with the addition amounts of 0.5, 1.0, 1.5, 2.0, and 2.5%. The polyamides were shaken at 28°C for 3 h and then filtered using suction. The resulting filtrate was scanned using an ultraviolet (UV) spectrophotometer from 190 to 800 nm. The absorbance of the highest peak, the polysaccharide content, and the protein content were recorded before and after purification. The polysaccharide retention rate, deproteinization rate, and decolorization rate were calculated using Equations (1), (2), and (3), respectively. Finally, the sample was dialyzed (7 kDa molecular weight cutoff dialysis bag) for 72 h for subsequent purification as follows:


(1) $$\mathrm{Polysaccharide}\mathrm{retention}\mathrm{rate}\left(\%\right)=\frac{A_{2}}{A_{1}}\times 100\%$$

(2) $$\mathrm{Deproteinization}\mathrm{rate}\left(\%\right)=\frac{\left({\text{B}}_{1}-{\text{B}}_{2}\right)}{{\text{B}}_{1}}\times 100\%$$

(3) $$\mathrm{Decolorization}\mathrm{rate}\left(\%\right)=\frac{\left({\text{C}}_{1}-{\text{C}}_{2}\right)}{{\text{C}}_{1}}\times 100\%$$

where A_1_ is the polysaccharide content before purification, A_2_ is the polysaccharide content after purification, B_1_ is the protein content before purification, B_2_ is the protein content after purification, C_1_ is the pigment scan absorbance before purification, and C_2_ is the pigment scan absorbance after purification.

• Standard curve creation and protein sample detection
• For standard curve creation and protein sample detection, standard samples of bovine serum albumin of different qualities were dissolved in 1 mL of ultrapure water. Then, 5 mL of Coomassie Brilliant Blue solution was added, with test tube 0 being used as a blank tube. The absorbance was measured at 595 nm, resulting in a standard curve (y = 0.007x − 0.0103, *R*^2^ = 0.9993) with a linearity range of 0–100 μg/mL. The protein content in the sample was determined with reference to the standard detection method comprehensive score for polyamide purification.

A comprehensive analysis of the deproteinization rate and polysaccharide retention rate of polyamide was performed using the following Equation:


(4) $$\text{I}=0.5\times \left(\frac{\text{X}}{{\text{X}}_{\text{max}}}+\frac{\text{Y}}{{\text{Y}}_{\text{max}}}+\frac{\text{Z}}{{\text{Z}}_{\text{max}}}\right)$$

where X is the deproteinization rate, Y is the polysaccharide retention rate, Z is the decolorization rate, X_max_ is the maximum deproteinization rate, Y_max_ is the maximum polysaccharide retention rate, and Z_max_ is the maximum decolorization rate, and 0.5 is a comprehensive coefficient.

(2) DEAE-52 cellulose ion exchange column for the second purification

• Cellulose preparation

As described by Huajie (33), 60.0 g of DEAE-52 cellulose was weighed, soaked in ultrapure water, boiled in a water bath for 30 min, and cooled to 25°C. Then, the upper layer of water was removed, and 0.5 mol/L sodium hydroxide solution was added before heating in a boiling water bath for 30 min. After cooling to room temperature, the upper layer of sodium hydroxide solution was removed, and the precipitate was washed with ultrapure water until the solution became neutral. Next, 0.5 mol/L hydrochloric acid solution was added, boiled in a water bath for 30 min, and cooled to 250 and cooled 30 acid solution was added, boiled in ntil the s, and the precipitate was washed with ultrapure water until the solution became neutral.

• Column equilibration, sample loading, elution, and collection

A chromatography column (2.6 × 60 cm) was installed vertically on an iron stand. After debugging the constant flow pump, the DEAE-52 cellulose wet method was applied to the column. Subsequently, the column was equilibrated with ultrapure water and injected with a constant flow pump. The preliminarily purified polysaccharide solution (5 mL) was slowly added to the chromatography column and eluted with NaCl solutions with concentrations of 0, 0.1, 0.3, 0.5, and 1 mol/L at a flow rate of 2 mL/min. Then, 10 ml was collected in each tube, and the absorbance of the eluent in each tube was measured at 490 nm using the phenol-sulfuric acid method. The resulting elution curve was drawn with the tube number as the abscissa and the polysaccharide absorbance as the ordinate. The eluates from each fraction were pooled, concentrated, and lyophilized.

(3) Sephadex G-100 for the third purification

For the final round of purification, 40.0 g of Sephadex G-100 was soaked in ultrapure water before heating in a 60°C water bath for 1 h until the Sephadex G-100 particles swelled. The water was replaced every 20 min, and the supernatant was discarded, followed by cooling to room temperature (34). Then, Sephadex G-100 was slowly added into a 2.6 × 60 cm column.

λ Column equilibration, sample loading, elution, and collection

The dried polysaccharide was dissolved after the second step of purification into 5 mL of pure water. This solution was slowly added to the above column and eluted with pure water at a flow rate of 1 mL/min. Then, 10 mL of each tube was collected, and the polysaccharide absorbance of the eluate in each tube was measured using the phenol-sulfuric acid method. The elution curve was drawn with the tube number as the abscissa and the polysaccharide absorbance as the ordinate. The fractional eluates were combined, concentrated, and lyophilized.

#### 2.2.2. Structural characterization of GFP

##### 2.2.2.1. Determination of monosaccharide composition

(1) Preparation and calculation of standard solutions

A total of 16 monosaccharide standards were used to obtain a standard stock solution: fucose (Fuc), rhamnose (Rha), arabinose (Ara), galactose (Gal), glucose (Glc), xylose (Xyl), mannose (Man), fructose (Fru), ribose (Rib), galacturonic acid (GalA), glucuronic acid (GlcA), galactosamine hydrochloride (GalN), glucosamine hydrochloride (GlcN), N-acetyl-D glucosamine (GlcNAc), guluronic acid (GulA), and mannuronic acid (ManA). Each monosaccharide standard solution was prepared with a precise concentration standard as the mixed standard. The masses of the different monosaccharides were measured according to the absolute quantitative method, and the molar ratio was calculated according to the molar mass of the monosaccharides.

(2) Sample preparation

The sample (5 mg) was placed in an ampoule bottle to which 2 mL of 3M TFA was added. This was then hydrolyzed at 120°C for 3 h. The acid hydrolysis solution was transferred to a tube, dried with nitrogen, and added 5 mL of water before vortexing. Then, 50 μL was pipetted and 950 μL of deionized water was added, followed by centrifugation at 11,279 × g for 5 min. The resulting supernatant was collected for ion chromatography (IC) (ICS5000; Thermo Fisher Scientific, United States) analysis.

##### 2.2.2.2. Chromatographic methods

Dionex Carbopac TM PA20 (3 × 150 mm) was used as the chromatographic column with the following mobile phases: A (H_2_O), B (15 mM NaOH), and C (15 mM NaOH and 100 mM NaOAC). A flow rate of 0.3 mL/min and an injection volume of 5 μL was applied. The column temperature was 30^°^C, and analysis was performed using a chemical detector.

##### 2.2.2.3. UV spectrophotometer scan

*Grifola frondosa* polysaccharides (GFPs) were scanned using a UV spectrophotometer at a scanning wavelength of 190–400 nm.

##### 2.2.2.4. Scanning electron microscopy scan

The freeze-dried samples were sprayed with gold and then scanned using scanning electron microscopy (SEM) (ZEISS 300; Germany) to photograph the morphology of the samples.

##### 2.2.2.5. Fourier transform infrared spectroscopy test

For the test, 2 mg of GFP and 200 mg of pure KBr were ground evenly, placed in a mold, pressed into a transparent sheet on a hydraulic press, and placed in an infrared spectrometer (NICOLET 670; United States). The wavenumber range was 4,000–400 cm^–1^, the number of scans was 32, and the resolution was 4 cm^–1^.

##### 2.2.2.6. X-ray diffraction scan

X-ray diffraction (XRD) (Bruker D8 Advance; Germany) was conducted using a copper target with a scanning range of 5–90° and a scanning speed of 10°/min.

##### 2.2.2.7. Thermogravimetric differential scanning calorimetry test

N_2_ was used as the test gas with a temperature range of 30–4,000°C, and a heating rate of 100f 30ento perform a comprehensive thermal analysis by thermogravimetric differential scanning calorimetry (TG-DSC) (TA SDT 600; United States).

##### 2.2.2.8. Establishment of the UC model

Based on previous studies (35, 36), we established an oxazolone (OXZ)-induced colitis model in mice. Mice were anesthetized with 2% sodium pentobarbital (3.5 μL/g). Then, their abdomens were shaved (2 × 2 cm), and a 3% oxazolone-methanol solution was applied (200 μL per mouse), which were air-dried after they appeared red and swollen. The application was repeated at an interval of 1 day, with the final application on the 5th day.

On the 7th day, a silicone tube was inserted 4 cm into the intestine through the anus. Then, 1% oxazolone-methanol solution was injected into the intestine, and the mouse was inverted for 30 s. The surgery was repeated on the 7th day, the 9th day, and after 11 days. On the 13th day, if the mice had loose stool or bloody stool, it was considered a successful model of enteritis.

##### 2.2.2.9. Mice grouping and treatment

Kunming mice (6–8 weeks old, 28–33 g) were obtained from the Experimental Animal Center of Guizhou Medical University, China. All procedures and experiments were approved by the Experimental Animal Ethics Committee of the Guizhou Medical University, China (approval no. 2200225).

A total of 72 mice (36 females and 36 males) were selected and randomly divided into experimental groups: model group (Model) (mice in the model group were killed after successful modeling) and the GFP-80 mg/kg/day (GFP-80), GFP-160 mg/kg/day (GFP-160), GFP-320 mg/kg/day (GFP-320), sulfasalazine 370 mg/kg/day (SASP) (37), and natural recovery (NR) groups. Another 12 healthy mice (6 females and 6 males) were placed in the control group (Control). The mice were treated by gavage with the above samples/drug for 7 consecutive days. After 7 days, the mice were killed, and samples were collected for analysis.

1) Record the weight change of each group of mice

At 3:00 p.m. every day, the mice were weighed, the data were recorded, and the average value of repeated weighing was recorded three times.

2) Colon length

After the mice were killed, their colon and intestine were measured with a ruler, and the colon length was recorded.

3) Determination of immune factors

After the mice were killed, colon tissues were collected and immediately stored in a refrigerator at −80°C. Before the experiment, the samples were thawed, homogenized using a tissue homogenizer at 2–8ti and centrifuged at 4°C and 4,000 rpm for 10 min. The concentrations of different cytokines (IL-1β, IL-10, and TNF-α) in the supernatants of the colon tissues were quantitatively determined using an enzyme-linked immunosorbent assay (ELISA) kit.

4) Routine blood index detection

Blood samples were collected from the inferior vena cava of mice after the intervention and placed in an EP tube pretreated with ethylenediaminetetraacetic acid (EDTA) to prepare whole blood. White blood corpuscles (WBC), red blood corpuscles (RBC), hemoglobin (HGB), platelet count (PLT), neutrophil (NEUT), and lymphocyte (L) counts were analyzed using an automatic hematology analyzer.

##### 2.2.2.10. Histological analysis

After colon tissue sampling, the colon was fixed with 4% paraformaldehyde at room temperature. After 24 h, the fixed colon tissues were embedded in paraffin. After embedding, the tissue was cut into 4-micron sections using a tissue biopsy machine and tested according to the instructions of the hematoxylin–eosin (HE) staining kit.

1) Statistical analysis and drawing

Statistical analysis was conducted using SPSS 20.0, High Score, GraphPad Prism 8, Adobe Illustrator 2020, Design-Expert 13, and Origin 2022. Differences were considered statistically significant at *P* < 0.05.

## 3. Results and discussion

### 3.1. Single-factor experiment

**FIGURE 1:**
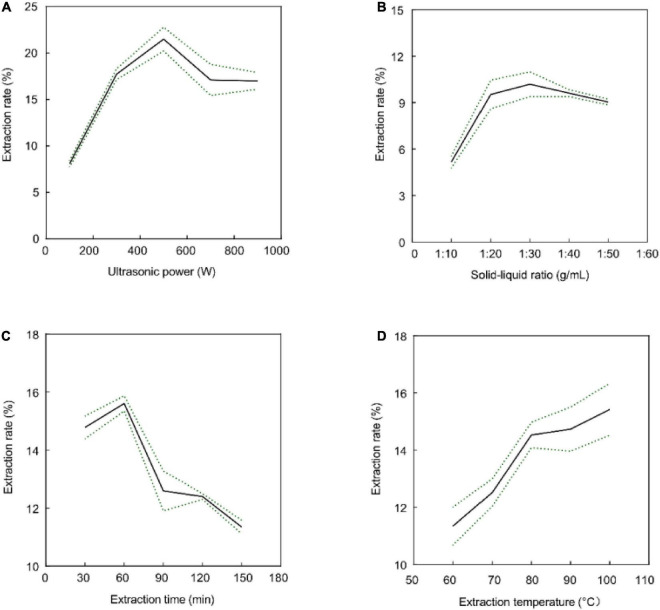
Effects of extraction time **(A)**, ultrasonic power **(B)**, solid-to-liquid ratio **(C)**, and extraction temperature **(D)** on the extraction rate of crude GFP. Data are expressed as the mean ± SD (*n* = 3). All the data between groups were statistically significant (*P* < 0.05).

Previous studies have shown that ultrasonic power, time, liquid-to-solid ratio, and temperature affect the extraction rate of polysaccharides (38–40). The effects of these parameters on the crude GFP extraction rate are shown in Figure 1. As shown in Figure 1A, the extraction time increased from 30 to 60 min, and the extraction rate was highest at 60 min; however, when the extraction time exceeded 60 min, polysaccharide degradation was induced, and the extraction rate decreased (41). In addition, the crude extraction rate of GFP increased with an increase in ultrasonic power in the range of 100–500 W, whereas the extraction rate began to decrease when the power was greater than 500 W (Figure 1B), possibly because the ultrasonic energy promoted the emptying formation of bubbles, the cell wall was destroyed, and the components began to dissolve. However, excessive ultrasonic power can result in structural changes or degradation of the polysaccharides (42). In addition, with an increase in the solid-to-liquid ratio from 1:10 to 1:30 (g/mL), the extraction rate of GFP was improved, and the extraction rate of crude GFP decreased after more than 1:30 (g/mL) (Figure 1C). This may be due to the high solid-to-liquid ratio, which resulted in a decrease in density and viscosity, which diluted the polysaccharides (43). Figure 1D shows that the extraction rate of crude GFP kept increasing at elevated temperatures; however, the increased extraction rate became slower after 80°C. In line with the principle of saving energy, we did not continue to increase the temperature. Ultimately, 80°C, 60 min, 1:30 (g/mL), and 500 W were identified as the optimal extraction conditions.

### 3.2. Crude BMF extraction method optimized by response surface methodology (RSM)

Based on the aforementioned results, we used the Boxid not continue ental design (BBD) to optimize the extraction conditions of crude GFP and summarized the BBD matrix and experimental data (Table 1). The final second-order polynomial equation, as shown in Equation 4, is obtained as follows:


(5) $$Y=14.11+5.47\,X_{1}+X_{2}+1.07\,X_{3}-0.38\,X_{4}-0.23\,X_{1}\,X_{2}-0.88\,X_{1}\,X_{3}-1.4\,X_{1}\,X_{4}-1.38\,X_{2}\,X_{3}+0.97\,X_{2}\,X_{4}-0.72\,X_{3}\,X_{4}+1.69\,X_{1}^{2}-0.64\,X_{2}^{2}-4.25\,X_{3}^{2}-2.09\,X_{4}^{2}$$

**TABLE 1:** BBD and results of crude GFP extraction experiments.

No.	X_1_ (extraction temperature, °C)	X_2_ (extraction time, min)	X_3_ (ultra- sonic power, W)	X_4_ (solid-to-liquid ratio, g/mL)	Y (extrac- tion rate, %)
1	1	−1	0	0	19.22
2	1	1	0	0	21.47
3	0	0	−1	−1	7.94
4	1	0	0	1	20.17
5	0	0	0	0	14.52
6	−1	0	1	0	8.47
7	0	0	0	0	13.53
8	0	1	1	0	10.35
9	−1	−1	0	0	8.47
10	−1	0	−1	0	4.11
11	−1	0	0	−1	5.05
12	−1	1	0	0	10.94
13	0	−1	1	0	10.94
14	0	1	0	−1	12.06
15	1	0	1	0	16.58
16	0	0	0	0	15.07
17	0	0	0	0	14.52
18	0	1	0	1	11.88
19	0	0	1	1	6.23
20	0	−1	−1	0	5.94
21	1	0	−1	0	15.76
22	0	−1	0	−1	12.17
23	0	0	0	0	12.93
24	1	0	0	−1	19.82
25	0	0	1	−1	10.99
26	0	−1	0	1	8.12
27	0	0	−1	1	6.05
28	−1	0	0	1	10.99
29	0	1	−1	0	10.88

In Equation 4 and table, Y represents the extraction rate, and X_1_, X_2_, X_3_, and X_4_ denote the extraction temperature, extraction time, ultrasonic power, and solid-to-liquid ratio, respectively.

Analysis of variance (ANOVA) of the quadratic polynomial model was fitted according to BBD (Table 2). The high model *F*-value (19.07) and very low *p*-value (< 0.0001) indicated that the model was very meaningful. At the same time, the lack of fit of the *F*-value (3.75) and *p*-value (0.1074) indicated that the relative error of this model was not significant. These results confirm the applicability and goodness of fit of the model to the predicted values (44).

**TABLE 2:** Analysis of variance results for the BBD models of extraction rate.

Source	Sum of squares	DF	Mean square	*F*-value	*P*-value
Model	590.38	14	42.17	19.07	<0.0001
X_1_	359.6	1	359.6	162.59	<0.0001
X_2_	12.04	1	12.04	5.44	0.0351
X_3_	13.82	1	13.82	6.25	0.0255
X_4_	1.76	1	1.76	0.79	0.388
X_1_X_2_	0.21	1	0.21	0.096	0.7616
X_1_X_3_	3.13	1	3.13	1.42	0.2538
X_1_X_4_	7.81	1	7.81	3.53	0.0812
X_2_X_3_	7.65	1	7.65	3.46	0.0841
X_2_X_4_	3.74	1	3.74	1.69	0.2142
X_3_X_4_	2.06	1	2.06	0.93	0.351
X_1_^2^	18.55	1	18.55	8.39	0.0117
X_2_^2^	2.62	1	2.62	1.18	0.295
X_3_^2^	117.04	1	117.04	52.92	<0.0001
X_4_^2^	28.44	1	28.44	12.86	0.003
Residual	30.96	14	2.21		
Lack of fit	27.98	10	2.8	3.75	0.1074
Pure error	2.99	4	0.75		
Cor total	621.34	28			
*R* ^2^	0.9502				
*R*^2^ *_Adj_*	0.9003				
CV	0.73%				
Adeq. precision	16.166				

X_1_, X_2_, X_3_, and X_4_ denote the extraction temperature, extraction time, ultrasonic power, and solid-to-liquid ratio, respectively.

#### 3.2.1. Analysis of response surface

**FIGURE 2:**
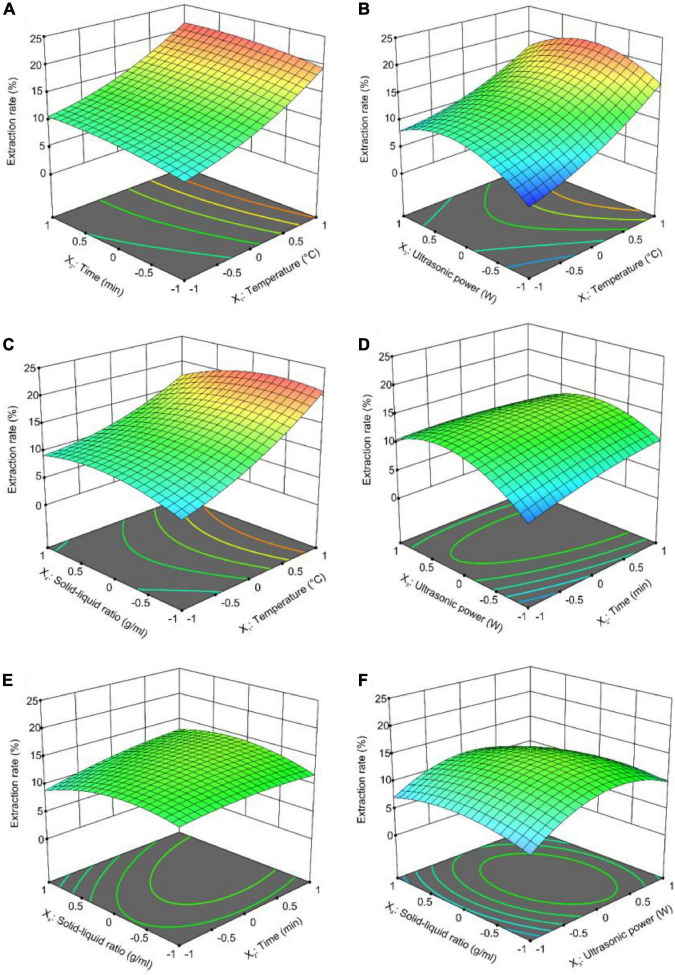
Response surface plots (3D) for the ultrasound-assisted extraction of crude GFP: **(A)** Extraction time vs. extraction temperature; **(B)** ultrasonic power vs. extraction temperature; **(C)** solid-to-liquid ratio vs. extraction temperature; **(D)** ultrasonic power vs. extraction time; **(E)** solid-to-liquid ratio vs. extraction time; and **(F)** solid-to-liquid ratio vs. ultrasonic power.

Design-Expert is a process widely used by researchers to optimize extraction conditions. It can predict the relationship between each influencing factor and the extraction rate and infer the best extraction rate of the method (45). In this study, crude GFP expression was affected by these four factors. In Figure 2, the relationship between the extraction rate of crude GFP and any two influencing factors (other factors were set to zero) is shown. The rounder the contour plot curve appeared in the response surface plot, the smoother the curve trend and the smaller the impact. As shown in Figure 2, the interaction between extraction time, extraction temperature, and the solid-to-liquid ratio were highly significant. The interaction between ultrasonic power and extraction temperature, ultrasonic power and solid-to-liquid ratio, ultrasonic power and extraction time were highly significant of the extraction rate. Solid-to-liquid ratio had no significant effection of the extraction rate of polysaccharides.

#### 3.2.2. Verification of the predictive model

The optimal process conditions obtained by RSM optimization were as follows: extraction time, 70.59 min; extraction temperature, 90°C; solid-to-liquid ratio, 1:36.58 g/mL; and ultrasonic power, 498.8 W. The theoretical value of the polysaccharide extraction rate was 21.72%. Considering the feasibility of practical operation, the optimal conditions for crude GFP extraction were revised to an extraction time of 71 min, an extraction temperature of 90°C, a solid-to-liquid ratio of 1:37 g/mL, and an ultrasonic power of 500 W. To test the accuracy of the RSM optimization results, crude GFP was extracted under the optimized conditions (*n* = 3). The results showed that the actual extraction rate was 21.13%, which was 0.59% different from the theoretical value. This indicates that the extraction process of crude GFP optimized by RSM is reliable and stable.

### 3.3. Purification of crude GFP

**FIGURE 3:**
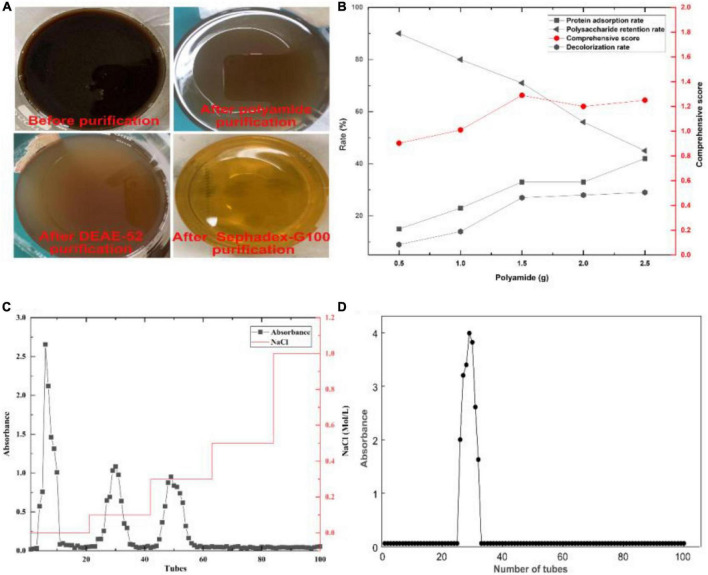
Crude GFP was purified using a three-step purification method. **(A)** Change in color during purification. **(B)** Changes in protein, color, polysaccharide, and comprehensive score during polyamide purification. **(C)** Three components were generated during the separation and purification of DEAE-52. **(D)** The elution result of GFP by Sephadex G-100.

After purification by polyamide, the color and protein of crude GFP were removed (Figures 3A, B). Since polyamide can adsorb certain polysaccharides, the comprehensive score was used as a unified evaluation method to remove protein and color while retaining the most polysaccharides. In addition, three polysaccharide fractions appeared after purification with DEAE-52 (Figure 3C), and the purification of the fraction with a higher extraction rate using Sephadex G-100 yielded a chart showing only one elution peak (Figure 3D).

The eluate in the mixed collection tube was concentrated and freeze-dried to obtain dry polysaccharides from which the polysaccharide content was determined. The results showed that the polysaccharide content was 92.50 ± 2.31%, indicating that the aforementioned purification had a good separation effect on crude GFP and that the obtained GFP had a high purity, providing a guarantee for the accuracy post-experiment.

### 3.4. Partial structural elucidation of GFP

**FIGURE 4:**
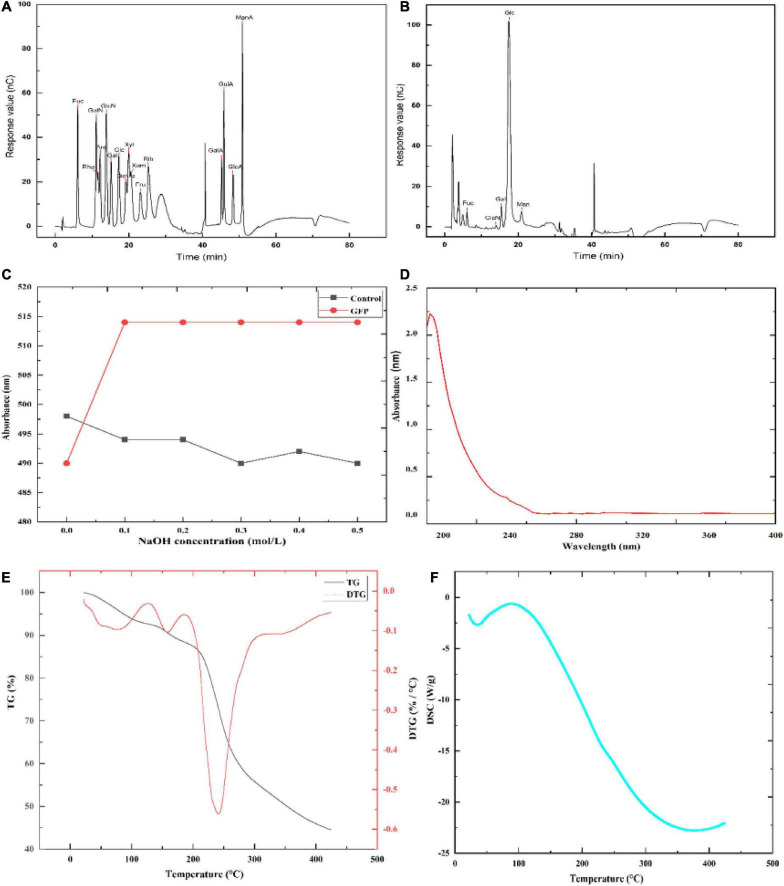
Partial structural analysis of GFP: **(A)** Ion chromatograms of 16 monosaccharide standards; **(B)** ion chromatograms of monosaccharides in GFP; **(C)** Congo red experimental result of GFP; **(D)** UV scan result of GFP; and **(E,F)** TG-DSC comprehensive thermal analysis results of GFP.

The monosaccharide composition of GFP is shown in Figure 4B and Table 3. By comparing the retention times of the standards (Figure 4A and Table 3), GFP was confirmed to be a glucan-type polysaccharide (46). After GFP formed a complex with Congo red, the maximum absorption wavelength was red-shifted compared with that of Congo red. When the NaOH concentration was 0.0–0.1 mol/L, the UV absorption shifted to the long wavelength (Figure 4C), indicating that the sample could form a complex with Congo red with a regular helical conformation. In other words, GFP formed an ordered three-dimensional helical structure in the weakly basic range. After UV scanning, no absorption peaks were observed at 260 nm and 280 nm, indicating that there were no nucleic acids or proteins in GFP (Figure 4D) (47). TG and DSC can be used to study the mass loss and thermal transitions during heating under an inert atmosphere. As shown in Figures 4E, F, the TG experiment resulted in two mass loss events for GFP, the first at 158.41°C, which may be attributed to the loss of adsorbed water and structural water of GFP, consistent with previous studies (48, 49). The DSC experiments showed that GFP absorbed 225.38 J/g of heat during an endothermic event around 89.78°C, most likely due to water evaporation, consistent with the TG analysis. The second mass loss with an initial temperature of 158.41°C and a peak temperature of 241.29°C resulted in a 39.458% reduction in the weight of the decomposed polysaccharides. The DSC experiment showed a good correlation with the TG peak temperature. The maximum thermal decomposition temperature of GFP was 241.29°C.

**TABLE 3:** Monosaccharide composition in GFP.

Name	Molar ratio of each monosaccharide in GFP
Fuc	0.025
GlcN	0.004
Gal	0.063
Glc	0.869
Man	0.038

Fuc, fucose; Gal, galactose; Glc, glucose; Man, mannose; GlcN, glucosamine hydrochloride.

**FIGURE 5:**
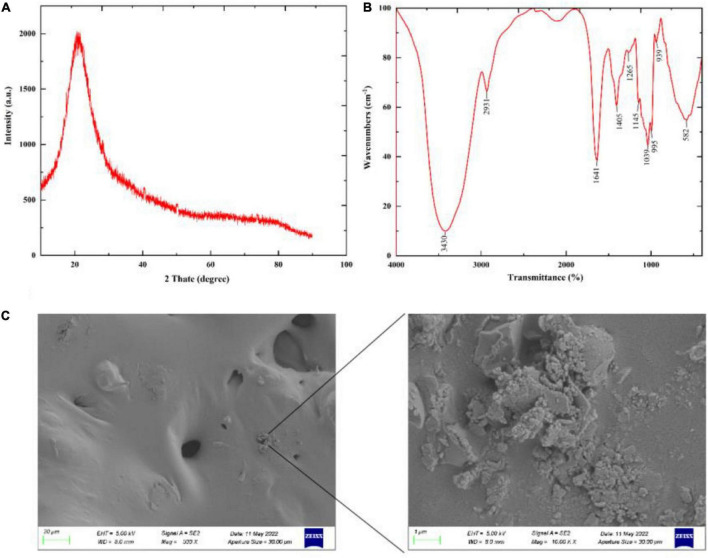
Partial structural analysis of GFP: **(A)** XRD scan result of GFP; **(B)** FTIR detection result of GFP; and **(C)** SEM detection result of GFP.

XRD can be used to determine whether a polysaccharide has a crystalline structure. As shown in Figure 5A, the XRD results showed that GFP was an amorphous or semi-crystalline substance with a “bun shape” structure and no sharp peak at 22.5°, which is consistent with the results of Zhang et al. (50). The Fourier transform infrared spectroscopy (FTIR) results are shown in Figure 5B. According to these results, characteristic peaks of carbohydrates were observed in the band range from 3,300 to 3,500 cm^–1^ (51), while a broad peak was observed at 3,430 cm^–1^, indicating the presence of O-H stretching vibrations (52). This suggests that GFP had typical carbohydrate characteristics. An absorption peak was also observed at 2,931 cm^–1^, attributed to C-H stretching vibration (53, 54), while an absorption peak around 1,600 cm^–1^ indicated that GFP had C = C vibration. Furthermore, 1,410 and 1,100 cm^–1^ were observed near the absorption peaks, indicating that there were C-H variable angle vibrations and C-O stretching vibrations in GFP (53). The absorption peak at 1,265 cm^–1^ was attributed to the C-O stretching of acetyl groups (55), while in the fingerprint region (1,400–650 cm^–1^), the absorption peak is usually related to the C-C and C-O stretching bonds and the bending vibration between C-H. A ß-glycosidic chain was observed in the sugar residue at 939 cm^–1^, indicating that GFP was mainly ß-type. In addition, characteristic peaks at 600id0 cm^–1^ indicated that GFP was a pyran-type polysaccharide (56). SEM analysis showed that most of the GFP was flat and contained a small number of particles (Figure 5C). This phenomenon may be explained by the existence of hydrogen bonds in polysaccharides (57). Due to the formation of a large number of hydrogen bonds between polysaccharide molecules, high-molecular-weight polysaccharides precipitate rapidly and tend to exhibit a relatively aggregated state.

### 3.5. Anti-inflammatory activity of GFP

**FIGURE 6:**
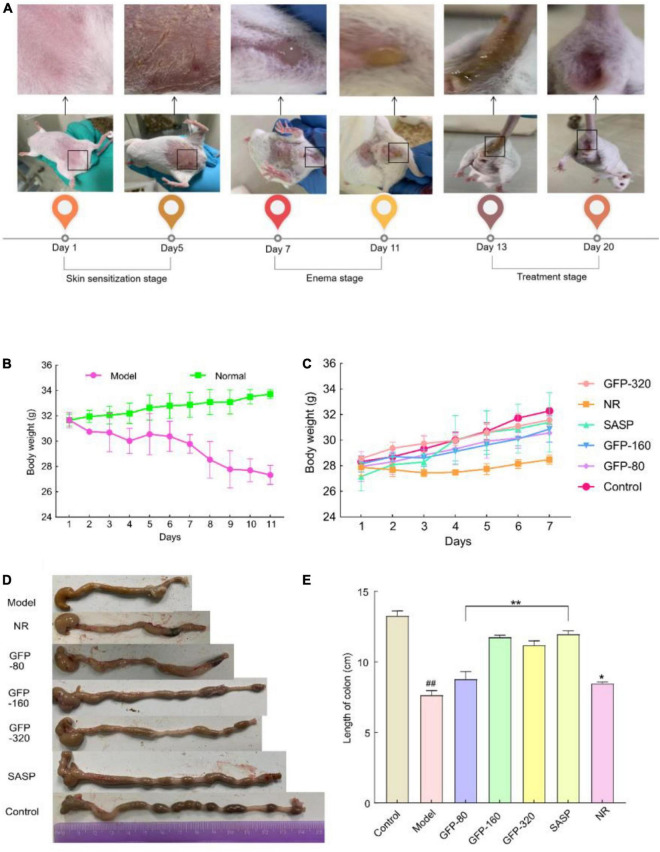
Changes in body weight and colon length of mice in each group. **(A)** Apparent changes in mice at different stages. **(B,C)** The body weight of mice at different stages. **(D,E)** Changes in the colonic length of mice at different stages. Each value represents the mean ± SD of six independent experiments. ^##^Very significant difference compared with the control group (*P* < 0.001); *significant difference compared with the model group (*P* < 0.05), **extremely significant difference compared with the model group (*P* < 0.001), * or ^#^no significant difference (*P* > 0.05).

Abdominal contamination and dehydration are typical symptoms of UC (58). Thus, changes in body weight are an important index for evaluating the success and effect of an acute colitis model (59). In this study, we observed that GFP treatment improved diarrhea and weight loss caused by OXZ, as shown in Figures 6A, B. During the modeling stage, the body weight of the mice decreased significantly due to severe UC. During the intervention stage (Figures 6A, C), the body weight of the mice in each group began to recover. However, weight gain in mice in the NR group was lower than that of mice in the GFP group. Colon shortening is another important manifestation of acute colitis; UC can cause congestion and edema, leading to colon shortening (59). As shown in Figure 6D, we removed and straightened the whole colon from the cecum to the anus and found that there was a statistically significant difference in colon length among the seven groups (*P* < 0.01) (Figure 6E). This suggests that GFP can significantly restore the colon length of OXZ-induced UC mice and that the symptoms of UC were alleviated earlier in the GFP intervention group than in the NR group. This was consistent with the changes in body weight.

The mechanism of UC is not fully understood and has been listed as a risk factor for the development of colorectal cancer. As a result, there is currently no specific effective drug for the treatment of UC (60). Intestinal mucosal immunity plays an important role in the occurrence and development of intestinal inflammation, and many components of the mucosal immune system, including lymphocytes and leukocytes, are closely related to the development of UC (61). Intact colonic mucosa also serves as a barrier to protection and the intestinal immune system (62). In our study, by observing the changes in the number of blood cells in blood routine indexes, we were able to evaluate the blood condition and conduct a preliminary screening for diseases. We also conducted routine blood tests in the experimental mice (Table 4) and found that the levels of WBC, PLT, NEUT, and L in the model group were significantly higher than those in the control group (*P* < 0.001). After GFP treatment, the levels of WBC, PLT, NEUT, and L in the GFP groups were significantly lower than those in the model group (*P <* 0.001). However, no significant differences were observed in the levels of HGB and RBC among the groups (*P* > 0.05). These results indicate that GFP could reduce WBC and other inflammatory indices and inflammatory lesions in UC model mice.

**TABLE 4:** Detection of blood routine indices in mice.

Index	Control	GFP-80	GFP-160	GFP-320	SASP	NR	Model
WBC/(10^9^/L)	2.41 ± 0.10	5.533 ± 0.26**	3.61 ± 0.15**	3.76 ± 0.20**	3.39 ± 0.18**	6.33 ± 0.24	6.70 ± 0.11^##^
RBC/(10^12^/L)	7.75 ± 0.16	7.70 ± 0.28	7.61 ± 0.32	7.35 ± 0.26	7.40 ± 0.33	7.54 ± 0.13	7.44 ± 0.15
HGB/(g/L)	139.37 ± 5.09	130.74 ± 8.56	127.67 ± 7.95	136.37 ± 12.44	140.63 ± 14.47	138.15 ± 7.17	136.67 ± 9.09
PLT/(10^9^/L)	550.00 ± 22.19	872.22 ± 14.19*	696.67 ± 16.82**	722.22 ± 21.69**	727.78 ± 20.58**	895.56 ± 23.12	902.22 ± 11.60^##^
NEUT/(10^9^/L)	0.41 ± 0.02	0.64 ± 0.02**	0.52 ± 0.03**	0.59 ± 0.01**	0.46 ± 0.01**	0.69 ± 0.02**	0.79 ± 0.02^##^
L/(10^9^/L)	1.56 ± 0.04	5.31 ± 0.12**	3.35 ± 0.33**	33.57 ± 0.05**	1.92 ± 0.02**	6.14 ± 0.11	6.18 ± 0.38^##^

Each value represents the mean ± SD of three independent experiments, ^##^very significant difference compared with the control group (*P* < 0.001); *significant difference compared with the model group (*P* < 0.05), **extremely significant difference compared with the model group (*P* < 0.001).

**FIGURE 7:**
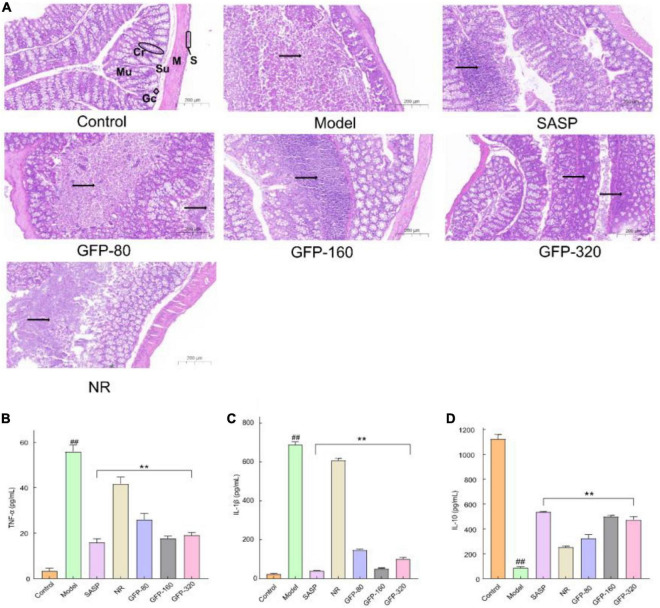
Results of histological examination and inflammatory factors in each group. **(A)** Results of histological examination. Mu, mucosa; Su, submucosa; M, muscle; S, serosa; Cr, crypt; Gc, goblet cell; →, inflammation. **(B)** The concentrations of TNF-α. **(C)** The concentrations of IL-1β. **(D)** The concentrations of IL-10. Each value presents the mean ± SD of three independent experiments. ^##^Very significant difference compared with the control group (*P* < 0.001), **extremely significant difference compared with the model group (*P* < 0.001).

Istological examination of the colon section (×200 μ m) is shown in Figure 7A. The structure of the colon in the control group was clear, while the mucous layer of the normal colon was filled with goblet cells. In the Model group, the basic structure of the colon was completely destroyed, and a large number of inflammatory cells infiltrated from the mucous layer to the muscular layer After the administration of SASP and GFP, the histological injury of the colon induced by OXZ was significantly alleviated, and the degree of remission was better than that of the NR group. A cross-section of the colon from the GFP-80 group showed a large amount of inflammatory cells infiltration and spread widely in the mucous membrane. Compared with the GFP-80 group, the therapeutic effects of the SASP, GFP-160, and GFP-320 groups were better, and only local inflammatory infiltration was observed in the mucosa of the colon tissue. In addition, the spread range of inflammatory cells in the GFP-160 group was smaller than that in the GFP-320 group, which showed that there was no dose-effect relationship between GFP and UC treatment.

According to the literature, the epithelial and immune cells in the intestines of the UC mice express a variety of inflammatory mediators (63). UC is associated with a decrease in the anti-inflammatory cytokine IL-10 and an increase in pro-inflammatory cytokines, such as IL-1 β and TNF-α (64, 65). When a large number of pro-inflammatory cytokines and chemokines are released into the tissue, B cells are actively secreted, produce antibodies, increase the humoral immune response, and stimulate the complement system, thereby triggering an inflammatory response (66). As an effective NF-κB activator, IL-1 β occurs in the early stages of intestinal inflammation and maintains the inflammatory environment in the colon (67). TNF-α is an effective pro-inflammatory cytokine that plays an important role in immune regulation, inflammatory response, proliferation, and death of all cell types (68). The level of TNF-α was often increased in the blood, fecal samples, and mucosa of patients with UC (69). The release of IL-10 can reduce the degree of the immune response, promote intestinal mucosal repair, and improve colonic inflammation. In this study, compared with the control group, IL-1 β and TNF-α in the OXZ group increased significantly, while the content of the anti-inflammatory cytokine IL-10 decreased significantly (Figures 7B–D). Treatment with GFP and SASP significantly enhanced the expression of the anti-inflammatory cytokine IL-10 and reduced the levels of pro-inflammatory cytokines (IL-6 and TNF-α). In addition, SASP and GFP-160 exhibited superior anti-inflammatory effects. These results further demonstrate the role of GFP in relieving OXZ-induced UC.

## 4. Conclusion

In summary, the amount of crude GFP obtained using ultrasonic-assisted extraction was 21.13% with a purification yield of 64.13%. The purity of purified GFP reached 92.50%. The polysaccharide composition was composed of the following molar ratios: fucose (0.025): glucosamine hydrochloride (0.004): galactose (0.063): glucose (0.869): mannose (0.038). After detection by XRD, FT-IR, and UV, GFP was found to be mainly β-glucan. In addition, GFP was found to inhibit colonic shortening caused by UC, reduce the secretion of pro-inflammatory factors, and increase the secretion of anti-inflammatory factors. These results suggest that GFP therapy could improve UC caused by ZOX and that the dietary polysaccharides found in *Grifola frondosa* could be used in the treatment of colitis in the future. Further studies will be needed to determine the specific changes in the structure of polysaccharides extracted by ultrasound-assisted extraction, as well as to better understand the occurrence, development mechanisms, and treatment mechanisms of UC to enable the development of specific therapeutic drugs.

## Data availability statement

The original contributions presented in this study are included in the article/Supplementary material, further inquiries can be directed to the corresponding authors.

## Ethics statement

This animal study was reviewed and approved by the Guizhou Medical University Laboratory Animal Ethics Committee, Guizhou Medical University.

## Author contributions

XL conceived and designed the experiments and wrote the manuscript. KH and SW revised the manuscript and performed visual analysis. SC, JX, and HL contributed to data processing and result analysis. XL and QZ conducted experiments and collected and organized data. SW and PL involved in conceptualization, supervision, project funding acquisition, supervision, and administration. All authors contributed to the article and approved the submitted version.
