# Supplementary material for: Structural properties and anti-inflammatory activity of purified polysaccharides from Hen-of-the-woods mushrooms (Grifola frondosa)
Source: Front Nutr. 2023 Feb 7;10:1078868. doi: 10.3389/fnut.2023.1078868 (PMC9941675; doi:10.3389/fnut.2023.1078868)
Supplement: Supplementary file 1 [file Presentation_1.pdf]

Supplementary Material

Structural Properties and Anti-Inflammatory Activity of Purified Polysaccharides from *Hen-of-the-Woods* Mushrooms (*Grifola frondose*

Shaofeng Wei*, Peng Luo* Xiaoyi Liu, Shuai Chen, Huijuan Liu, Jiao Xie, K. M. Faridul Hasan, Qibing Zeng

*** Correspondence:**
Shaofeng Wei
shaofenggy@163.com

Peng Luo

luopeng@gmc.edu.cn

Supplementary Figures

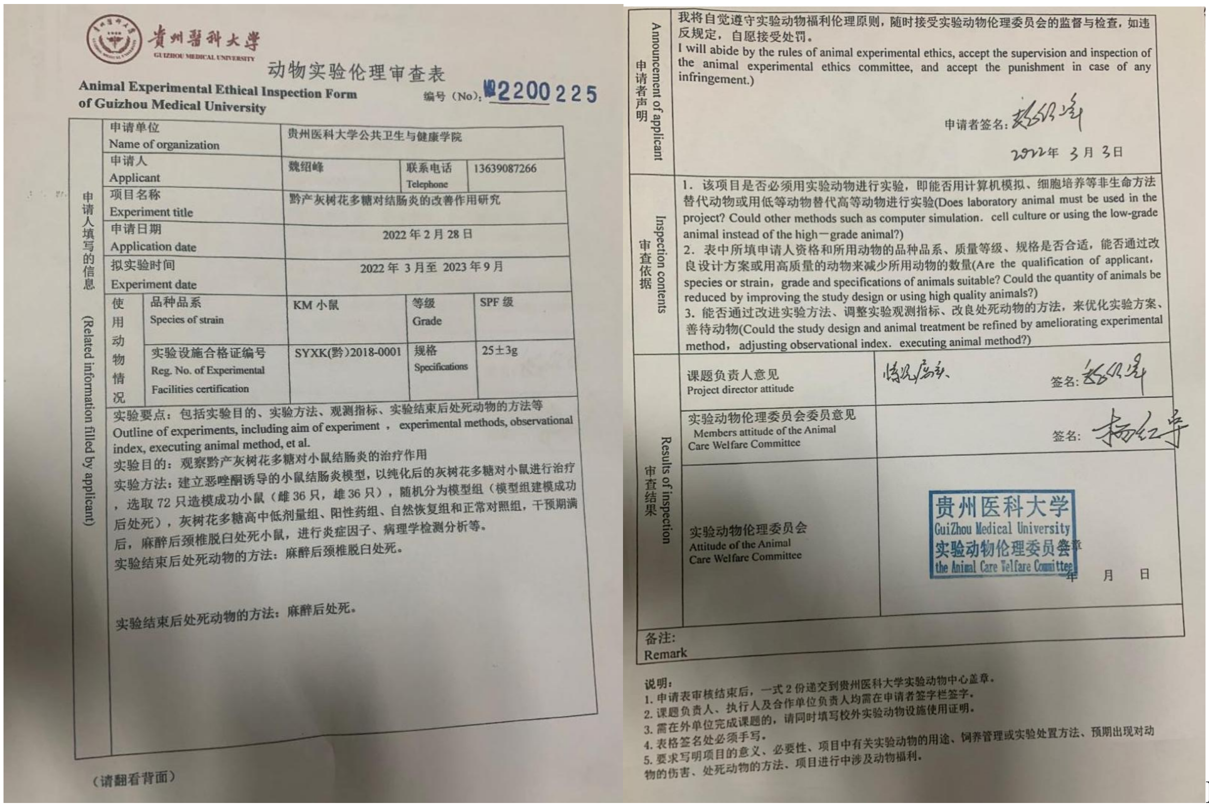
贵州医科大学
GUIZHOU MEDICAL UNIVERSITY
动物实验伦理审查表
Animal Experimental Ethical Inspection Form of Guizhou Medical University
编号（No）: 2200225

申请人填写的信息 (Related information filled by applicant)

申请单位 Name of organization	贵州医科大学公共卫生与健康学院		
申请人 Applicant	魏绍峰	联系电话 Telephone	13639087266
项目名称 Experiment title	黔产灰树花多糖对结肠炎的改善作用研究		
申请日期 Application date	2022 年 2 月 28 日		
拟实验时间 Experiment date	2022 年 3 月至 2023 年 9 月		
使用动物情况 品种品系 Species of strain	KM 小鼠	等级 Grade	SPF 级
实验设施合格证编号 Reg. No. of Experimental Facilities certification	SYXK(黔)2018-0001	规格 Specifications	25±3g

实验要点：包括实验目的、实验方法、观测指标、实验结束后处死动物的方法等
Outline of experiments, including aim of experiment ， experimental methods, observational index, executing animal method, et al.
实验目的：观察黔产灰树花多糖对小鼠结肠炎的治疗作用
实验方法：建立恶唑酮诱导的小鼠结肠炎模型，以纯化后的灰树花多糖对小鼠进行治疗，选取 72 只造模成功小鼠（雌 36 只，雄 36 只），随机分为模型组（模型组建模成功后处死），灰树花多糖高中低剂量组、阳性药组、自然恢复组和正常对照组，干预期满后，麻醉后颈椎脱臼处死小鼠，进行炎症因子、病理学检测分析等。
实验结束后处死动物的方法：麻醉后颈椎脱臼处死。

实验结束后处死动物的方法：麻醉后处死。

（请翻看背面）

申请者声明 Announcement of applicant
我将自觉遵守实验动物福利伦理原则，随时接受实验动物伦理委员会的监督与检查，如违反规定，自愿接受处罚。
I will abide by the rules of animal experimental ethics, accept the supervision and inspection of the animal experimental ethics committee, and accept the punishment in case of any infringement.)
申请者签名：
2022年 3月 3日

审查依据 Inspection contents
1．该项目是否必须用实验动物进行实验，即能否用计算机模拟、细胞培养等非生命方法替代动物或用低等动物替代高等动物进行实验(Does laboratory animal must be used in the project? Could other methods such as computer simulation．cell culture or using the low-grade animal instead of the high－grade animal?)
2．表中所填申请人资格和所用动物的品种品系、质量等级、规格是否合适，能否通过改良设计方案或用高质量的动物来减少所用动物的数量(Are the qualification of applicant, species or strain，grade and specifications of animals suitable? Could the quantity of animals be reduced by improving the study design or using high quality animals?)
3．能否通过改进实验方法、调整实验观测指标、改良处死动物的方法，来优化实验方案、善待动物(Could the study design and animal treatment be refined by ameliorating experimental method，adjusting observational index．executing animal method?)

审查结果 Results of inspection
课题负责人意见 Project director attitude　签名：
实验动物伦理委员会委员意见 Members attitude of the Animal Care Welfare Committee　签名：
实验动物伦理委员会 Attitude of the Animal Care Welfare Committee　贵州医科大学 GuiZhou Medical University 实验动物伦理委员会 the Animal Care Welfare Committee　章　年　月　日

备注: Remark

说明：
1. 申请表审核结束后，一式 2 份递交到贵州医科大学实验动物中心盖章。
1. 申请人、执行人及合作单位负责人均需在申请者签字栏签字。
2. 课题负责人、执行人及合作单位负责人均需在申请者签字栏签字。
2. 课题负责人、执行人及合作单位负责人均需在申请者签字栏签字。

Figure S1. Proof of ethical approval.

Supplementary Tables

Table S1. Extraction rate and components of GFP.

	Polysaccharide samples	
	**Crude GFP**	**GFP**
Rate of extraction (%)	21.72	63.65
Chemical composition		
Protein (%)	18.23 ± 0.04	0
Polysaccharide (%)	49.62 ± 0.08	92.50 ± 2.31
Polyphenols (%)	5.77 ± 0.01	0

Table S2. Molecular weights of purified GFP

Parameter	Mw	Mp	Mn	Mz	Mz+1	Mv
Value (Da)	2.565×10^5	2.997×10^5	2.059×10^5	3.031×10^5	3.438×10^5	2.494×10^5
